# Non-Pharmaceutical Interventions against COVID-19 Causing a Lower Trend in Age of LHON Onset

**DOI:** 10.3390/genes14061253

**Published:** 2023-06-12

**Authors:** Yuxi Zheng, Xiaoyun Jia, Shiqiang Li, Xueshan Xiao, Qingjiong Zhang, Panfeng Wang

**Affiliations:** 1 State Key Laboratory of Ophthalmology, Zhongshan Ophthalmic Center, Sun Yat-sen University, Guangdong Provincial Key Laboratory of Ophthalmology and Visual Science, 54 Xianlie Road, Guangzhou 510060, China; zhengyx29@mail2.sysu.edu.cn (Y.Z.); lishiqiang@gzzoc.com (S.L.); xiaoxueshan@gzzoc.com (X.X.); 2 Gene Diagnostic Laboratory, Genetic Eye Clinic, Zhongshan Ophthalmic Center, Sun Yat-sen University, 54 Xianlie Road, Guangzhou 510060, China

**Keywords:** COVID-19, Leber hereditary optic neuropathy, m.11778G>A, age of onset, risk factors

## Abstract

Leber hereditary optic neuropathy (LHON) is a monogenic but multifactorial disease vulnerable to environmental triggers. Little is known about how LHON onset changed during the COVID-19 pandemic and how non-pharmaceutical interventions (NPHIs) against COVID-19 impact LHON onset. One hundred and forty-seven LHON patients with the m.11778G>A mutation complaining of vision loss were involved between January 2017 and July 2022. The onset time points, age of onset, and possible risk factors were evaluated. Analyses were conducted among 96 LHON patients in the Pre-COVID-19 group and 51 in the COVID-19 group. The median (IQR) age of onset decreased significantly from 16.65 (13.739, 23.02) in pre-COVID-19 to 14.17 (8.87, 20.29) during COVID-19. Compared with the Pre-COVID-19 group, the COVID-19 group exhibited bimodal distribution with an additional peak at six; the first quarter of 2020 also witnessed a relatively denser onset, with no subsequent second spike. NPHIs against COVID-19 significantly changed patients’ lifestyles, including higher secondhand smoke exposure (*p* < 0.001), adherence to masks (*p* < 0.001), reduction in time spent outdoors for leisure (*p* = 0.001), and prolonged screen time (*p* = 0.007). Multivariate logistic regression revealed that secondhand smoke exposure and mask-wearing were independent risk factors of younger LHON onset. Lower age of onset of LHON appeared after the breakout of the COVID-19 pandemic, and novel risk factors were detected, including secondhand exposure and long mask-wearing. Carriers of LHON mtDNA mutations, especially teenagers or children, should be advised to avoid secondhand smoke exposure and there are possible adverse outcomes of longer mask-wearing.

## 1. Introduction

Leber hereditary optic neuropathy (LHON, OMIM #535000) is the most common form of mitochondrial disease in young adults, with the peak age of onset preferentially in the second and third decades of life, and this coincides with the fact that more than 90% of affected Chinese carriers develop the disease younger than 35 [1,2]. Although the pathogenic mtDNA mutations are homoplastic or nearly homoplastic in most LHON pedigrees, the penetrance can vary among different families carrying the same mutation and even within the same family among other branches, which cannot be due to mtDNA mutations alone [3,4]. The discordant onset in two pairs of monozygotic twins and better prognosis in childhood-onset LHON further supported the fact that environmental modifiers interact with the primary pathogenic mtDNA variants and thus regulate the incomplete penetrance of LHON [5,6].

Since the breakout of the COVID-19 pandemic, unprecedented life-altering effects have been brought on adults and children. The implementation of strict non-pharmaceutical interventions (NPHIs) to contain COVID-19, including mask-wearing, school and workplace closure, and home quarantine, has been reported to be associated with the onset or progression of complex diseases, such as a boom in myopia [7], exacerbation in diabetes [8], and increase in preterm stillbirth [9]. A similar situation was also observed in older sporadic patients whom LHON attacked because of increased alcohol consumption or smoking during the COVID-19 pandemic [10]. So, we carried out this retrospective study to systemically study the effect of lifestyle changes caused by COVID-19 on the onset of LHON and to find possible novel risk factors. On the other hand, it cannot be neglected that the risk factors of certain diseases are distinct across human populations with different genetic backgrounds [11]. Therefore, most of the research that investigates the effects of risk factors related to NPHIs on complex diseases might be limited since the genetic backgrounds of most of them were hard to consider [12,13]. LHON, a monogenic but multifactorial disease, acts as an ideal disease model to investigate the effect of COVID-19 containment measures on the disease when the participants were under controllable genetic differences [14].

Herein, we conducted a cohort study of LHON patients with the m.11778G>A mutation to assess the changes in LHON onset during the COVID-19 pandemic and try to uncover the potential risk factors for penetrance.

## 2. Materials and Methods

### 2.1. Study Design and Population

Patients complaining of sequentially bilateral, painless, subacute visual failure onset from January 2017 to July 2022 and who were genetically diagnosed with the mtDNA m.11778G>A mutation using Sanger sequencing were included. Before peripheral blood was collected for the genetic test, the probands or their legal guardians signed written informed consent. This study conformed to the tenets of the Declaration of Helsinki, and ethical approval was obtained from the institutional review board of Zhongshan Ophthalmic Centre, Guangzhou, China.

The symptom onset time was divided into two phases: pre-COVID-19 (January 2017 to December 2019), corresponding to the period prior to the COVID-19 breakout, and COVID-19 (January 2020 to July 2022), corresponding to the period in which social restrictions were still in place.

### 2.2. Data Collection

Detailed disease history of all participants, including the age, gender, time of vision loss, previous treatment, family history, and residence, was obtained by senior ophthalmologists during the first visit. Potential risk factors were selected based on literature reviews [10,15]. A quantitative questionnaire survey via phone or in an outpatient clinic was used to collect reliable patient information between July 2022 and September 2022. Socioeconomic characteristics prior to symptom onset included education level, occupation, and annual family income (CNY). Clinical-related and lifestyle indicators before the vision loss included alcohol consumption and smoke exposure, mask-wearing habits, COVID-19 vaccination, daily screen time, and outdoor activity time. Secondhand smoke is defined as the parents or any other family members living with the patients smoking at home [16]. Participants are allocated to the secondhand smoke exposure if there is at least one smoker at their home; those who are nonsmokers and have no smoker at home are classified as non-smoke exposed. Outdoor time comprises time spent outdoors for sports and tome spent outdoors for leisure (walking to and from school, post-dinner walking, playing in the park) [17]. Daily digital screen time consists of time spent on TV, computer, smartphone, and tablet. Based on the assumption that a non-response phone call—defined as no answer after ten calls, a dead number, or a redirection of calls, is entirely random, non-answered phone call participants were excluded for deeper analysis.

### 2.3. Statistical Analysis and Visualization

Statistical analysis and visualization were performed in open source R (version 4.1.2) and RStudio. Categorical variables are displayed as numbers and the different prevalence between groups was analyzed using a Chi-square test or Fisher’s exact test. Continuous variables that passed the normality test are presented as mean (SD) and were assessed using Student’s *t*-test; otherwise, the Mann–Whitney test was used. Statistical significance was defined as *p* < 0.05. Sociodemographic characteristics and COVID-19-related pressures that were assessed as clinically relevant or those with a significant level of *p* < 0.05 in univariate analysis were included for the multivariate logistic regression model. Given the rarity of LHON and the number of outcome events, the final model was constructed by carefully limiting the number of variables included.

## 3. Results

### 3.1. Comparison of LHON Onset between the Pre-COVID-19 Group and the COVID-19 Group

**Figure 1:**
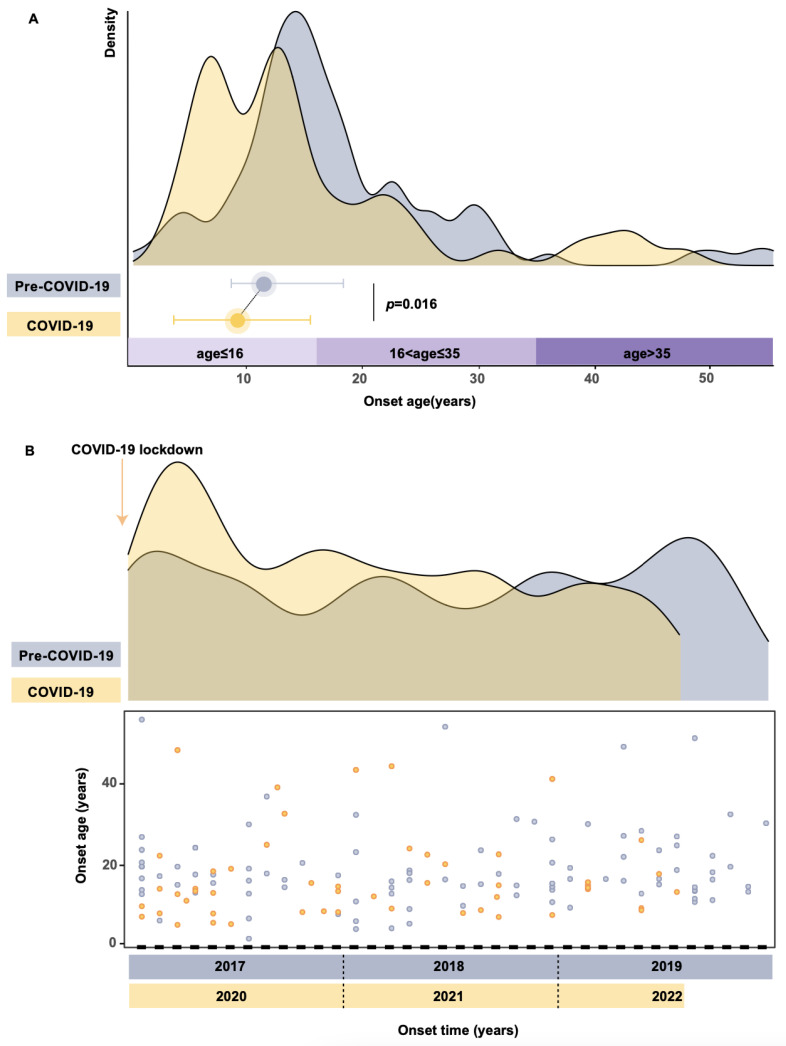
Distribution of LHON onset at different time points and ages. (**A**) Quantification of age of onset in the Pre-COVID-19 group (blue) and the COVID-19 group (yellow). The color bar on the horizontal axis represents the three-age of onset groups. Blue and yellow dots indicate the median age of onset of pre-COVID-19 and COVID-19, respectively. The Kernel density curve was generated and arranged using the R package ggridges and ggplot2 with adjust equal to 0.5. (**B**) A combined scatter plot and histogram of LHON onset in the pre-COVID-19 era (blue) and the COVID-19 era (yellow). The horizontal axis represents the time (months) and the vertical axis is the age of onset. Each dot represents the timepoint and age of onset of individuals when experiencing sudden vision loss. Each year was divided into 12 segments on the *x*-axis.

There were 147 patients with the m.11778G>A mutation complaining of vision loss between January 2017 and July 2022. The median age of onset of LHON significantly decreased to 14.17 (8.97, 20.29) in the COVID-19 group compared to 16.65 (13.73, 23.02) in the Pre-COVID-19 group (*p* = 0.016), in accordance with the median age of onset of 16 in our prior research (Figure 1A) [2]. Unexpectedly, in addition to the forward shift of the age range from 16.65 to 14.17, a novel and pronounced peak at six occurred, different from previous observations [18]. To further reveal the underlying reason for this phenomenon, we illustrated the frequency of symptom onset at the different times between the two groups using kernel density plots associated with scatter plots in Figure 1B. No regular trend of LHON onset throughout the different time points was observed in the Pre-COVID-19 group (blue curve). The yellow density curve of LHON onset in the COVID-19 group had developed an elevation since January 2020, the beginning of the COVID-19 lockdowns imposed, which peaked three months later and then fell rapidly; no subsequent second COVID-19-associated spike was seen. The median age of onset declined significantly to 11.30 (7.79, 18.31) from January to March 2020 (early pandemic), with a Hodges–Lehmann median estimation difference of 5.356 (95% CI 0.3781–9.553) (*p* = 0.040) when compared with that in the Pre-COVID-19 group (January 2017 to December 2019). Box-violin plots indicated that seasonal variations had no statistically significant effect on LHON age of onset (Figure S1). The distribution difference among three age of onset groups in two periods shown in Table S1 achieved statistical significance (*p* = 0.001).

Notably, the male-to-female ratio of the probands in the whole cohort was 15.38:1 (139:8), with 23.00:1 (92:4) for the Pre-COVID-19 group and 11.75:1 (47:4) for the COVID-19 group (Table S1). When the affected family members were included in the analysis, the male-to-female ratio was 6.76:1 (115:17) for the Pre-COVID-19 group and 3.47:1 (59:17) for the COVID-19 group.

### 3.2. Socio-Demographic Characteristics of Study Patients

To further analyze how the NPHIs altered the age of onset, we retroactively interviewed patients about their lifestyles. Because up to 97% of the patients in this study manifested vision failure before they were 35 years old, consistent with the dominant age range in our prior research and a previous study on Chinese probands, we then focused on the patients with an age of onset of younger than 35 years [2,19]. Patients were divided into four groups according to the onset time being before or after COVID-19 and using a cut-off point of 16 years old, which is the end of the nine-year compulsory education according to China’s Compulsory Education law [2]: teenager-onset in pre-COVID-19 (TO-pre, onset ≤ 16 years), teenager-onset in COVID-19 (TO-post, onset ≤ 16 years), adult-onset in pre-COVID-19 (AO-pre, 16 < onset ≤ 35 years), and adult-onset in COVID-19 (AO-pre, 16 < onset ≤ 35 years). Socio-demographic characteristics and COVID-19-related characteristics were then compared among the four groups.

Out of the 137 participants with an age of onset younger than 35 years, 114 finished the phone or field questionnaire, a response rate of 83.21%, with a median age of 15.16 (11.70, 19.17) at onset. The 114 interviewed participants and the complete 137 participants were not different in any of the general socio-demographics (Table S2), indicating that loss during follow-up was less likely to affect the overall conclusions. The general characteristics and COVID-19-related lifestyle of the interviewed patients in two time periods are demonstrated in Table 1. Among the 114 participants, 73 were from pre-COVID-19, and 41 were from COVID-19, the median ages of onset of which were 16.53 (13.77, 21.37) and 13.22 (8.61, 15.67) (*p* < 0.001), respectively. The significant difference in age of onset also led to the differences in education (*p* < 0.001) and occupation (*p* = 0.013). Males were roughly 93.86% overall, with a male-to-female ratio of 15.28:1 based on probands and 5.12:1 based on affected family members. Most participants were students (72.80%) and reported an annual family income of less than 80,000 CNY (81.58%). The median education years of the non-students were 9.00 (9.00, 11.00), mostly with secondary education. There was no statistical difference in gender, geographical location, residence, the season of onset, family yearly income, and alcohol consumption between pre-COVID-19 and COVID-19 (Table 1).

### 3.3. COVID-19-Related Characteristics

As a monogenic multifactorial disease, LHON is susceptible to environmental factors. Since the outbreak of COVID-19, people’s lifestyle has been modified by interventions to oppose the pandemic, such as mask usage, home quarantine, and COVID-19 vaccination. Therefore, patients or guardians were asked about wearing a mask when going out, smoke exposure, daily screen time, and outdoor activity time, including outdoors for sports and outdoor for leisure, to investigate the intervention of NPHIs in LHON onset. Significant increases in secondhand smoke exposure (*p* < 0.001), adherence to masks (*p* < 0.001), prolonged screen time (*p* = 0.007), and decreased time spent outdoors for leisure (*p* = 0.001) were observed when comparing the COVID-19-related changes between the two periods (Table 1). Therefore, further analysis focused on these four potential risk factors.

**Figure 2:**
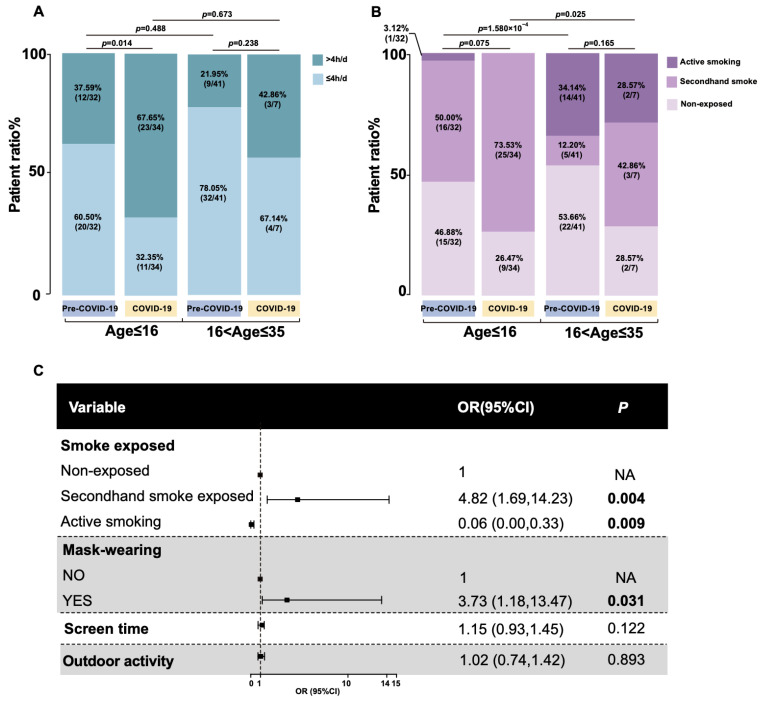
Uncovering potential risk factors for teenager-onset LHON. (**A**) Stack bar plot showing the proportion of digital screen time between the period before and after the COVID-19 pandemic categorized by teenager-onset group and adult-onset group. (**B**) Stack bar plot showing the proportion of smoke-exposed between the period before and after the COVID-19 pandemic categorized by teenager-onset group and adult-onset group. (**C**) Forest plot for putative triggers. Odds and 95% (CI) were computed and visualized using R package forestplot (version 3.1.1). OR, odds ratio.

Time spent on screen was dichotomized at 4 h/d, the median in the total questioned population. There was no statistical difference in TO-pre or TO-post when compared to the adult-onset group of the same time point, respectively (*p* = 0.488) (*p* = 0.673) (Figure 2A). It is also noteworthy that the duration of time spent outdoors for leisure in the COVID-19 group decreased to 0.50 (0.50, 0.75) h/day (*p* = 0.001), probably due to the policy of reduction in nonessential activity.

Prolonged screen time and limited outdoor leisure time collectively indicate a longer time at home and might increase the risk of secondhand exposure, especially for those whose family members are smoking. A higher prevalence of secondhand smoke exposure was observed in TO-pre or TO-post when, respectively, compared with the AO-pre and AO-post (*p* < 0.001) (*p* = 0.025), indicating teenagers had more secondhand smoke exposure (Figure 2B).

We distinguished those who reported wearing a mask when going out from those not reporting this preventive strategy. All the participants in the COVID-19 group reported wearing a mask, as mask usage is a cost-effective measure to alleviate the risk of COVID-19 transmissions. After adjusting for other potentially confounding factors, the impact of mask usage finds a significant effect on teenager-onset LHON (3.73; 95%CI, 1.18–13.47) (*p* = 0.031), and secondhand smoke was associated with nearly a five-fold increased risk of teenager-onset LHON with high heterogeneity (4.82; 95%CI, 1.69–14.23) (*p* = 0.004) (Figure 2C). As tobacco is banned from being sold to minors in China, only one of the juveniles in this study smoked; that is why active smoking was calculated as negatively associated with teenager-onset LHON.

For those who developed the disease after the COVID-19 breakout, we additionally inquired about COVID-19 vaccination and the date of doses given. Among the 19 patients who reported they were vaccinated, the coverage rate was 47.06% (16/34) in the TO-post group, while it was 42.86% (3/7) in the AO-post. There was no statistical difference in COVID-19 vaccination between the TO-post and AO-post (*p* = 0.839).

## 4. Discussion

Our study reports an increase in teenager-onset LHON after the breakout of COVID-19, with a proportion of 68.63% (35/51). The bimodal distribution of the COVID-19 group is observed with two peaks of nearly equal altitude, roughly at age six and thirteen. Even though childhood-onset LHON is correlated with a better prognosis when a higher spontaneous visual recovery rate was indicated, complete restoration of vision is not yet observed, and many of these teenagers sustain permanent vision impairment [6]. Therefore, it is of critical importance to uncover the risk factors behind this phenomenon. In the present study, the detailed retrospective investigation of the lifestyle habits allowed us first to propose secondhand smoke exposure and mask-wearing as potential triggers of teenager-onset LHON.

Several extrinsic factors have already been proposed for LHON onset, including gender, haplogroup, cigarette smoke, alcohol consumption, head injury, excessive blood loss, acute illness, and anti-retroviral and anti-tuberculosis drugs [20,21]. Even though cigarette smoke has been long recognized as a significant environmental trigger of LHON by reducing the copy number of mtDNA, most studies explored the influence of proactive instead of passive smoke [15]. Secondhand smoke is a significantly higher risk factor for teenager-onset LHON after adjustment for other potential triggers. Very scarce association between secondhand smoke exposure and ocular adverse effect has been reported, including refractive error, AMD, choroidal thinning, and cataracts; advocating hazards of environmental smoke should also be taken into consideration in ophthalmological perspectives in future studies [22,23]. Research conducted in Hong Kong indicated secondhand smoke exposure was associated with thinner peripapillary retinal fiber layers, which was a sensitive biomarker of axonal damage and the damage level of retinal ganglion degeneration [16]. Moreover, the concentration of secondhand smoke in the air is intense, especially in a confined space during the home quarantine, and teenagers have more narrow airways and inhale faster, making teenagers themselves more susceptible to environmental hazards [24].

According to a large cross-sectional study among 1745 individuals in mainland China, the mask-wearing rates were 20 of 1745 individuals (1.1%) before the COVID-19 pandemic, while during the COVID-19, the mask-wearing rate increased to 1090 of 1097 (99.4%) when the Chinese government launched a mandatory mask wearing policy in January 2020 [25]. It is reported that modest changes in healthy individuals’ gas exchange, pulmonary function, and psychological effects showed up after wearing a mask during exercising [26]. The subtle cerebral hemodynamics and oxygenation changes brought by daily mask usage raise a concern about exacerbation in the dysfunction of mitochondria in LHON carriers, as LHON is sensitive to mitochondrial biogenesis alteration and oxidative stress [27]. So, it is rational to treat mask-wearing as a candidate risk factor for LHON during COVID-19. Further analysis is needed to ensure that mask usage associated with teenager-onset LHON does not persist beyond the state of the COVID-19 pandemic.

The first spike in the time–density curve of LHON onset was in accordance with the mass quarantine in early 2020. Our figures show that the change in the age of LHON onset is not subject to seasonal variation, we suggest that the teenager-onset LHON boom is likely to be an effect of the COVID-19 home quarantine. Individuals who have been exposed to secondhand smoke may have already encountered this exposure from cohabitants prior to the COVID-19 pandemic; however, due to the prolonged time spent at home during lockdown measures, the intensity of secondhand smoke exposure might have increased, leading to the accumulation of smoke to a critical level. Additionally, changes in lifestyle practices, such as wearing a mask, might cause damage before potential protective pathways have had time to reach their full potential, leading to the onset of illness. As surgical masks are primarily designed to prevent the release of droplets, including saliva or respiratory droplets, from the wearer to others, instead of filtering airborne particles, such as the fine particulate matter found in secondhand smoke, it is possible that the combined effects of secondhand smoke exposure during home confinement and the potential impact of prolonged mask usage on oxygen intake may play a role in triggering LHON onset [28]. The following slight fluctuation reflects the neuroplasticity as restrictions were gradually eased, consistent with this hypothesis and a previous study on cortical plasticity linked to retinal ganglion cell loss [29,30]. Most of the teenagers in our study might be genetically primed to adult-onset or even lifelong asymptomatic LHON but became symptomatic early in life due to passive smoking and mask usage.

However, it should be noted that there were still 22.73% (15/66) of the teenager-onset LHON in our study neither exposed to secondhand smoke nor mask-wearing. The complex scenario between different environmental triggers and mtDNA haplogroup or even modifiers in nuclear genes requires further exploration. Although increased digital screen time and reduced outdoor time were revealed to be insignificantly associated with teenager-onset LHON, whether these short-term changes will persist or deteriorate remains to be determined as strict containment measures are still imposed to mitigate the spread of COVID-19. It has been reported that the burden of ophthalmic risk factors was higher among those of lower socioeconomic status, despite the accessibility of health care services [31,32]. There are about 81.6% (93/114) of patients in our cohort with a lower family income, and 62.3% (71/114) living in rural areas.

The overall prevalence of COVID-19 vaccination rate in the COVID-19 group is 46.34% (19/41), with 42.86% (3/7) in AO-post and 47.06% (16/34) in TO-post, the rate of which is lower than the first dose vaccine coverage rate of 98.02% observed in the general population in China [33]. Chief factors leading to vaccination hesitancy might be fears caused by some unscientific opinions such as vaccinations causing disease or deterioration of vision, especially for the mutation carriers and those with a family history of LHON. Our research indicates that COVID-19 vaccination was not a risk factor for earlier LHON onset, and the reasons lie in two factors: (1) The declined age of onset of LHON mainly happened from January to March 2020 when the COVID-19 vaccines were unavailable. (2) There was no statistical difference in COVID-19 vaccination between TO-post and AO-post. Given the efficacy of COVID-19 vaccines to lessen the hospitalization with COVID-19 pneumonia and the mortality rate [34], whether the LHON unaffected carriers or patients should refuse the vaccine or not remains conservative before a much larger sample size shows evidence of an increased risk for LHON onset after vaccination. According to the results of our questionnaire, none of the 147 individuals were infected with COVID-19 at the time of the survey. This is comparable to the cumulative number of confirmed COVID-19 cases reported in the general population in China, which stood at 250,449 by the end of September 2022, as documented by the China Center for Disease Control and Prevention (http://www.nhc.gov.cn/xcs/xxgzbd/gzbd_index.shtml, accessed on 9 June 2023), with an estimated COVID-19 infection rate of 0.17%, based on the seventh national census (https://www.gov.cn/guoqing/2021-05/13/content_5606149.htm, assessed on 9 June 2023). The COVID-19 infection was not treated as an independent risk factor in this study because none of the enrolled individuals were infected with the COVID-19 virus before September 2022. The influence of the COVID-19 infection on LHON onset will be based on data from December 2022 when China’s massive COVID-19 outbreak peaked in our future study.

Still, our study has some limitations. First, our study is limited by its retrospective nature and its associated recall bias. However, the rarity of LHOH makes it unrealistic to evaluate a more significant number of LHON patients prospectively. Second, the rarity of LHON imposes restrictions on the size of our cohort. However, the number of patients in our different groups satisfied the demand for statistical analysis. Third, it is likely that some of the LHON patients in the COVID-19 group might have been influenced by factors that happened before COVID-19. Almost all of the recent studies on extra abnormality onset or progression of several complex diseases during COVID-19 set lockdowns as the cut-off point when groups were concerned [17,25,35]. Some of them also pointed out the limitation that it is difficult to estimate the standpoint since the exact duration of exposure is unclear [35,36].

In summary, to minimize influences caused by different genetic backgrounds, we used the most common point mutation m.11778G>A of LHON as the study subject to reveal that the primary age of LHON onset shifted to an earlier time during the COVID-19 pandemic. These findings were associated with increased secondhand smoke exposure and mask-wearing habits, which should have a practical role in clinical implications and genetic counseling. For LHON carriers, secondhand smoke should also be strongly avoided, in addition to avoiding proactive smoking and efforts to relieve the potential adverse effects of mask-wearing should be considered.

## Figures and Tables

**Table 1:** Sociodemographic characteristics of patients with LHON**.**

Characteristics	Patients, No. (%) (*n* = 114)			*p* Value
	Overall$(*n* = 114)	Pre-COVID-19 $(*n* = 73)	COVID-19 $(*n* = 41)	
**Sociodemographic characteristics**				
**Age of onset, median (IQR)**	15.16 (11.70, 19.17)	16.53 (13.77, 21.37)	13.22 (8.61, 15.67)	**<0.001**
**Age of onset group, %**				
age ≤ 16	66 (57.89)	32 (43.84)	34 (82.93)	**<0.001**
16 < age ≤ 35	48 (42.11)	41 (56.16)	7 (17.07)	
**Gender**				
Male	107 (93.86)	68(94.44)	38 (92.68)	0.989
Female	7 (6.14)	4(5.56)	3 (7.32)	
**Annual family income, %**				
≤80,000 RMB	93 (81.58)	60 (82.19)	33 (80.49)	0.822
>80,000 RMB	21 (18.42)	13 (17.81)	8 (19.51)	
**Geographical location, %**				
North China	3 (2.63)	2 (2.74)	1 (2.44)	>0.999
South China	111 (97.37)	71 (97.26)	40 (97.561)	
**Residence, %**				
Rural areas	71 (62.28)	46 (63.01)	25 (60.98)	0.989
Urban areas	43 (37.72)	27 (37.99)	16 (39.02)	
**Season of onset, %**				
Cold season	50 (43.86)	30 (41.10)	20 (48.78)	0.44
Warm season	64 (56.14)	43 (58.90)	21 (51.22)	
**Education years, median (IQR)**	7.50 (6.00, 9.00)	9.00 (7.00, 10.0)	6.00 (2.00, 8.00)	**<0.001**
**Occupation, %**				
Student	83 (72.81)	47 (64.38)	36 (87.80)	**0.013**
Non-student	31 (27.19)	26 (35.62)	5 (12.20)	
**COVID-19 related pressures**				
**Time spent outdoors**	1.50 (0.75, 2.50)	1.75 (0.75, 3.00)	1.00 (0.50, 2.25)	0.074
Outdoors for sports, median (IQR), h/day	1.00 (0.00, 2.00)	1.00 (0.00, 2.00)	0.50 (0.00, 1.50)	0.583
Outdoors for leisure, mean (SD), h/day	0.75 (0.50, 1.00)	0.75 (0.50, 1.00)	0.50 (0.50, 0.75)	**0.001**
**Time spent on screen-based devices, h/day**	4.00 (2.00, 5.00)	3.00 (2.00, 5.00)	5.00 (3.00, 6.00)	**0.007**
**Smoke exposed**				
Firsthand smoke	17 (14.9)	15 (20.5)	2 (4.88)	**<0.001**
Secondhand smoke	49 (43.0)	21 (28.8)	28 (68.3)	
Non-exposed	48 (42.1)	37 (50.7)	11 (26.8)	
**Alcohol consumption**				
YES	13 (11.4)	10 (13.7)	3 (7.32)	0.372
NO	101 (88.6)	63 (86.3)	38 (92.7)	
**Vaccination**				
YES	-	-	19 (46.34)	0.839
NO	-	-	22 (53.66)	
**Mask-wearing habits**				
YES	-	0 (0.00)	41 (100.00)	**<0.001**
NO	-	73 (100.00)	0 (0.00)	

## Data Availability

The data presented are available on request from the corresponding author. The data are not publicly available due to ethical privacy.

## Supplementary Materials

The following supporting information can be downloaded at: https://www.mdpi.com/article/10.3390/genes14061253/s1, Figure S1: Box-violin plots for age of onset of LHON in four seasons before and during COVID-19; Table S1: Socio-demographic characteristics of all patients with LHON onset from January 2017 to July 2022; Table S2: Comparison of the demographic characteristics of patients with LHON onset younger than 35 years between the complete dataset and data from interviewed participants.
