# Supplementary material for: Non-Pharmaceutical Interventions against COVID-19 Causing a Lower Trend in Age of LHON Onset
Source: Genes (Basel). 2023 Jun 12;14(6):1253. doi: 10.3390/genes14061253 (PMC10298648; doi:10.3390/genes14061253)
Supplement: Supplementary file 1 [file genes-14-01253-s001.zip › 10.ZhengYX-Supplemantory table s2.pdf]

Supplementary table s2. Comparison of the demographic characteristics of patients with LHON onset younger than 35 years between the complete dataset and data from interviewed participants.

Characteristics	Patients, No.(%)								
	Overall			Pre-COVID-19			COVID-19		
	Complete (n=137)	Interviewed (n=114)	p.value	Complete (n=91)	Interviewed (n=73)	p.value	Complete (n=46)	Interviewed (n=41)	p.value
**Onset age,median (IQR)**	15.60 (12.33;20.62)	15.16 (11.70;19.17)	0.486	16.51 (13.50;20.66)	16.53 (13.77;21.37)	0.767	13.55 (8.67;16.36)	13.22 (8.61;15.67)	0.692
**Onset age group**									
age≤16	76 (52.41)	66 (57.89)	0.700	41 (45.05)	32 (43.84)	0.876	35 (76.09)	34 (82.93)	0.432
16<age≤35	61 (47.59)	48 (42.11)		50 (54.95)	41 (56.16)		11 (23.91)	7 (17.07)	
**Gender**									
Male	135 (95.07)	107 (93.86)	0.672	87(95.60)	68(94.44)	0.733	43 (93.48)	38 (92.68)	>0.999
Female	7 (4.93)	7 (6.14)		4(4.40)	4(5.56)		3 (6.52)	3 (7.32)	
**Residence**									
Rural areas	96 (67.61)	71 (62.28)	0.374	62 (68.13)	46 (63.01)	0.492	31 (67.39)	25 (60.98)	0.533
Urban areas	46 (32.39)	43 (37.72)		29 (31.87)	27 (37.99)		15 (32.61)	16 (39.02)	
**Geographical location**									
North China	4 (2.92)	3 (2.63)	>0.999	3 (3.30)	0 (0.00%)	0.255	1 (2.17)	1 (2.44)	>0.999
South China	133 (97.08)	111 (97.37)		88 (96.70)	73 (100.00%)		45 (97.83)	40 (97.56)	
**Season of onset**									
Cold season	66 (48.18)	50 (43.86)	0.526	44 (48.35)	30 (40.10)	0.431	22 (47.83)	20 (48.78)	>0.999
Warm season	71 (51.83)	64 (56.14)		47 (51.65)	43 (58.90)		24 (52.17)	21 (51.22)	
