# Supplementary material for: Non-Pharmaceutical Interventions against COVID-19 Causing a Lower Trend in Age of LHON Onset
Source: Genes (Basel). 2023 Jun 12;14(6):1253. doi: 10.3390/genes14061253 (PMC10298648; doi:10.3390/genes14061253)

Supplementary Figure S1. Box-violin plots for onset age of LHON in four seasons before and during the COVID-19.

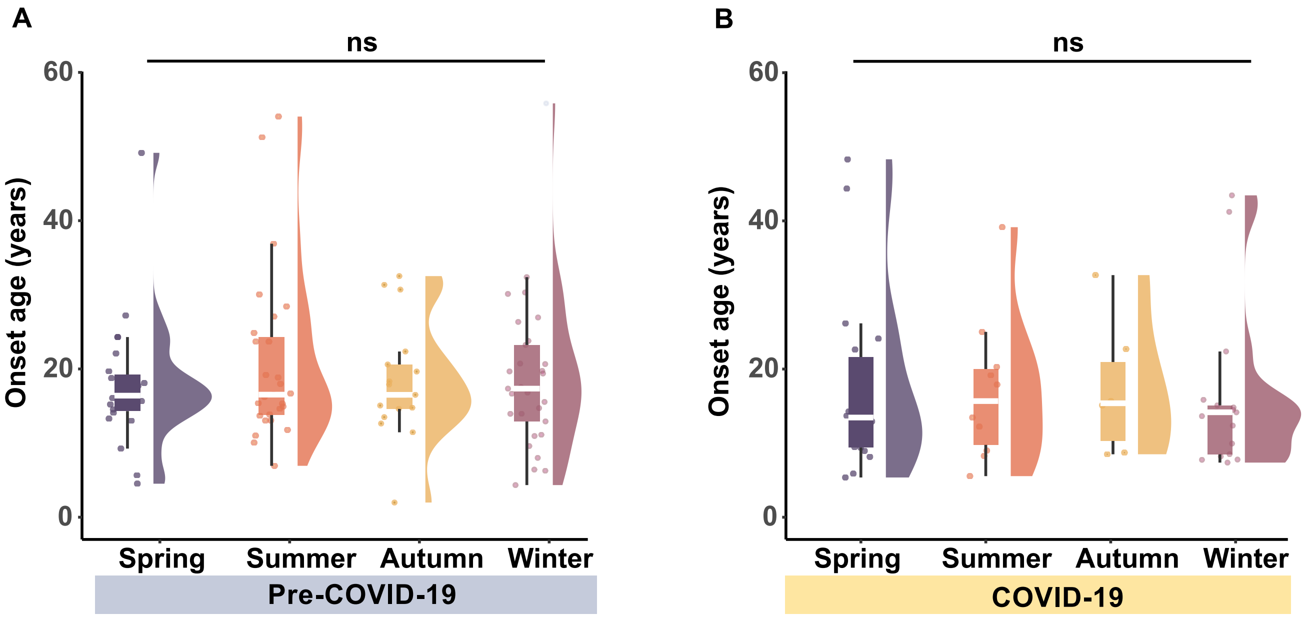

Supplement: Supplementary file 1 [file genes-14-01253-s001.zip › 8.ZhengYX-COVID-19-Supplementary figure s1.pdf]
