# Supplementary material for: Non-Pharmaceutical Interventions against COVID-19 Causing a Lower Trend in Age of LHON Onset
Source: Genes (Basel). 2023 Jun 12;14(6):1253. doi: 10.3390/genes14061253 (PMC10298648; doi:10.3390/genes14061253)
Supplement: Supplementary file 1 [file genes-14-01253-s001.zip › 9.ZhengYX-Supplemantory table s1.pdf]

Supplementary table s1. Scio-demographic characteristics of all patients with LHON onset from January 2017 to July 2022

Characteristics	Patients, No.(%)			p.value
	Overall (n=147)	Pre-COVID-19 (n=96)	COVID-19 (n=51)	
**Onset age,median (IQR)**	15.68 (12.64;22.35)	16.65 (13.73;23.02)	14.17 (8.97;20.29)	**0.016**
**Onset age group**				
age≤16	76 (51.70)	41 (41.67)	35 (68.63)	**0.001**
16<age≤35	61 (41.50)	50 (53.12)	11 (21.57)	
age>35	10 (6.80)	5 (5.21)	5 (9.80)	
**Gender**				
Male	139 (93.9)	92(94.44)	47 (92.68)	0.058
Female	8 (6.14)	4(5.56)	4 (7.32)	
**Geographical location**				
North China	143 (97.28)	93 (96.88)	50 (98.00)	0.999
South China	4 (2.72)	3 (3.12)	1 (2.00)	
**Residence**				
Rural areas	99 (67.35)	65 (67.71)	34 (66.67)	0.989
Urban areas	48 (32.65)	31 (32.29)	17 (33.33)	
**Season of onset**				
Cold season	71 (48.30)	45 (46.88)	26 (50.98)	0.729
Warm season	76 (51.70)	51 (53.12)	25 (49.02)	
